# Multi-Technique Characterization of Historic Blue Bricks from Beijing: Compositional Grouping, Weathering Assessment, and Conservation Implications

**DOI:** 10.3390/ma19122666

**Published:** 2026-06-21

**Authors:** Zhaoyang Zhu, Rui Hu, Bo Zhang

**Affiliations:** 1 School of Architecture and Art, North China University of Technology, Beijing 100144, China; 2 Beijing Institute of Archaeology, Beijing 100071, China

**Keywords:** historic blue bricks, multi-technique characterization, compositional classification, weathering assessment, heritage conservation

## Abstract

Historic blue bricks are fundamental to Beijing’s architectural heritage, yet cross-site compositional data for guiding material-compatible restoration remain scarce. This study applies WD-XRF, XRD, SEM, thermal expansion measurement, and physical property testing to 21 blue brick specimens from four Beijing-area sites spanning the Tang through Qing dynasties, with PCA and K-means clustering used to explore compositional grouping structures. Within this exploratory dataset, a compositional distinction separates the Ming and Qing Great Wall bricks: CaO falls from 7.7 to 1.5 wt.% as anorthite gives way to albite, while Qing specimens are denser (1.79 vs. 1.65 g·cm^−3^) with lower water absorption (15.9% vs. 20.9%). Two Wanping City bricks are strongly sulfate-enriched (SO_3_ up to 9.8%), and WP-SE3 additionally carries a heavy chloride load (Cl 2.1%), masking their original clay signatures and illustrating how unrecognized weathering can distort compositional grouping and source-related interpretation from bulk chemistry. K-means clustering yields compositional types that overlap only partially with site boundaries, capturing raw material variation rather than site-specific manufacturing fingerprints. Despite constraints in sample size and physical property coverage, the integrated dataset offers preliminary compositional benchmarks and limited performance data to inform period-specific brick replacement at these heritage sites.

## 1. Introduction

Historic blue bricks constitute the primary structural material of traditional Chinese architecture, from imperial palaces and city walls to Buddhist temples and military fortifications [1,2]. In this paper, “blue bricks” refers to qingzhuan, the grey-blue fired-clay bricks produced under a reducing atmosphere that are traditional to Chinese architecture; the terms “blue bricks” and “grey bricks” are used interchangeably in the literature for these reduced-atmosphere fired-clay bricks, and we use “blue bricks” consistently throughout. In Beijing, the capital of successive Chinese dynasties, blue bricks from multiple historical periods coexist within standing structures and archeological contexts, reflecting diverse raw material sources, production technologies, and administrative procurement systems spanning over a millennium [3,4]. The scientific characterization of these materials is essential for two complementary purposes: understanding the technological evolution of historical brick production, and providing compositional reference data to support material-compatible restoration practices in heritage conservation [5,6,7].

Recent advances in the analysis of architectural ceramics have made it increasingly feasible to characterize large numbers of brick specimens using X-ray fluorescence (XRF), X-ray diffraction (XRD), and scanning electron microscopy (SEM) [8,9,10,11,12,13]. XRF provides elemental composition of the bulk clay matrix, controlled primarily by the geological source of raw materials [14,15]. XRD reveals the mineralogical phase assemblage, sensitive to firing temperature and atmosphere [16,17,18,19]. SEM offers microstructural characterization at the micron scale, revealing grain morphology, porosity, and vitrification degree [20,21,22]. Physical property measurements—including bulk density, water absorption, and compressive strength—provide essential performance indicators directly relevant to durability assessment and conservation material selection [19,21].

A critical methodological challenge in brick provenance studies is the conversion of multi-element compositional data into meaningful group structures. Multivariate statistical methods—particularly principal component analysis (PCA) and cluster analysis—have been increasingly applied to archeological ceramic and brick datasets [9,10,23,24,25,26]. Zhang et al. (2025) [27] recently proposed an innovative “dual-channel” approach for inscriptionless blue bricks, combining K-means clustering of both XRD pattern data and XRF elemental data, and cross-referencing the two sets of results through a cross-matrix diagram. Applied to 42 Ming–Qing blue bricks from four Chinese sites, their method identified four characteristic brick types.

The present study draws on the XRF-based clustering approach of Zhang et al. (2025) [27] but does not replicate their full dual-channel methodology (which additionally requires clustering of complete XRD diffractogram data); instead, XRD information is used qualitatively for mineral phase identification and firing technology interpretation. Our study focuses on intra-regional variation among four Beijing-area sites representing different architectural functions (military defense, religious, civic) and historical periods (Tang through Qing). We address three questions: (1) What are the compositional, mineralogical, and physical property characteristics of blue bricks from each site? (2) Do bricks from different sites and periods form compositionally distinguishable groups? (3) Can the SiO_2_–CaO geochemical relationship identified by Zhang et al. be confirmed in an independent Beijing brick dataset?

## 2. Materials and Methods

### 2.1. Samples

A total of 21 blue brick samples were collected from four historic sites in the Beijing area (Table 1). The Great Wall samples (*n* = 8) comprise four Ming dynasty bricks (GW-M1–M4, dimensions approximately 44.4 × 21.9 × 9.6 cm) and four Qing dynasty bricks (GW-Q1–Q4, approximately 36.3 × 17.7 × 8.8 cm) from watchtowers at the Miyun section. The Lingyue Temple samples (*n* = 4) include two Tang dynasty bricks (LY-T1, LY-T2; LY-T2 measuring 38.6 × 19.5 × 6.7 cm). The Tang attribution of LY-T1 is independently supported by a thermoluminescence age of 1210 ± 120 yr BP (Beijing Hanzhen Ceramics Technology Co., Ltd., Beijing, China, report no. BJHZ1138; see Supplementary Table S1). The Lingyue Temple set also includes one brick from the west wall of the Tianwang Hall (LY-TWD) and one from the south sill wall of the Mahavira Hall (LY-DXB). The Zhengang Tower samples (*n* = 5) are from Buddhist niche structures at various vertical positions. The Wanping City samples (*n* = 4) are from the southeast and northeast corner towers of Wanping, a walled garrison town built in the late Ming dynasty on the western approach to Beijing, immediately east of the Lugou (Marco Polo) Bridge. All samples were obtained from broken or detached fragments during recent conservation work. With the exception of LY-T1, whose age was determined by thermoluminescence (TL) dating, the chronological attributions are based on archeological and typological assessment of brick characteristics by site specialists rather than on absolute dating; the attributions for the Zhengang Tower bricks and the later Lingyue Temple elements (LY-TWD, LY-DXB) should therefore be regarded as provisional. A sample-by-sample summary of site, architectural element, sampling context, and the basis and confidence of each chronological attribution is provided in Supplementary Table S1.

### 2.2. X-Ray Fluorescence (XRF)

Elemental compositions were determined using a Panalytical Axios (Malvern Panalytical B.V., Almelo, The Netherlands) wavelength-dispersive X-ray fluorescence (WD-XRF) spectrometer with an Rh target X-ray tube. Samples were cut, oven-dried at 105 ± 5 °C for 24 h, and ground to <75 μm. Analysis employed Panalytical SuperQ 5 software (Malvern Panalytical B.V., Almelo, The Netherlands) with Omnian semi-quantitative fundamental parameter procedures (25 kV, 96 mA). Results are reported as semi-quantitative oxide weight percentages (wt.%) from fundamental parameter calculations. The residual category “Other” listed in Supplementary Table S4 was calculated as 100% minus the sum of the listed oxides and minor elements and represents volatile components (H_2_O, CO_2_, SO_2_), trace elements below quantification limits, and analytical uncertainty; because loss on ignition (LOI) was not measured independently, “Other” should not be interpreted quantitatively as LOI. No certified reference materials were analyzed and no replicate measurements were performed for this dataset; accordingly, the XRF results are treated as semi-quantitative screening data suitable for comparative compositional grouping rather than for absolute geochemical certification. Nine oxides were detected above quantification limits in at least part of the sample set: SiO_2_, Al_2_O_3_, Fe_2_O_3_, CaO, K_2_O, MgO, Na_2_O, TiO_2_, and SO_3_. For multivariate analysis, six major oxides (SiO_2_, Al_2_O_3_, Fe_2_O_3_, CaO, K_2_O, MgO) were selected as the primary variable set, following established practice in clay provenance studies. In Table 2, values reported as n.d. (not detected) indicate concentrations below the instrument quantification limit. For the primary 6-oxide PCA and K-means analyses, only the six major oxides consistently detected in all 21 samples were used, so n.d. values do not affect these results. For the extended 9-oxide sensitivity analysis, n.d. values for TiO_2_ and SO_3_ were replaced with half the estimated quantification limit (0.3 wt.% for TiO_2_, 0.2 wt.% for SO_3_). A sensitivity test substituting n.d. with zero yielded virtually identical PCA loadings and cluster assignments, confirming that the n.d. treatment does not materially affect the results. An extended 9-oxide model (adding Na_2_O, TiO_2_, SO_3_) was also evaluated to assess the influence of additional elements on PCA and clustering outcomes (Section 3.7).

### 2.3. X-Ray Diffraction (XRD)

**Figure 1:**
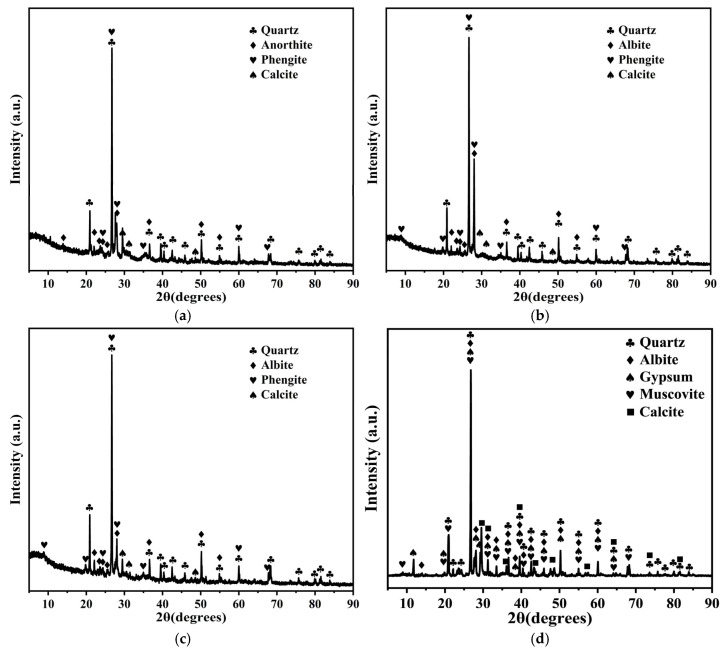
Representative XRD diffractograms with annotated mineral phase peaks: (**a**) GW-M1, Ming dynasty (Type A: Qz + An + Phg + Cal); (**b**) GW-Q1, Qing dynasty (Type B: Qz + Ab + Phg + Cal); (**c**) LY-T1, Tang dynasty (Type B: Qz + Ab + Phg + Cal); (**d**) WP-SE3, weathered Wanping City brick (Type B with Gypsum: Qz + Ab + Gyp + Ms + Cal). Although the patterns in (**b**,**c**) appear visually similar, they correspond to different raw data files (GW-Q1 and LY-T1, respectively); the principal distinction between Type A and Type B is the presence of anorthite versus albite, with diagnostic reflections annotated. WP-SE3 is shown as the representative Type B* pattern; WP-SE2 was assigned to Type B* based on the same diagnostic gypsum reflection. Reference patterns (PDF cards) for quartz, anorthite, albite, phengite, and calcite are provided in Supplementary Figure S1.

Mineralogical compositions were determined using a Panalytical Empyrean (Malvern Panalytical B.V., Almelo, The Netherlands) powder diffractometer with Cu Kα radiation (λ = 1.5406 Å; 40 kV, 40 mA). Divergence and scattering slits were each 1°, receiving slit 0.15 mm. Scans covered 2θ = 10–90° at 10°/min. Mineral phase identification used JADE 6 software (Materials Data, Inc., Livermore, CA, USA) with the ICDD PDF-4+ database. Representative diffractograms for four samples spanning the major compositional types (GW-M1, GW-Q1, LY-T1, and WP-SE3) are presented with annotated peak assignments (Figure 1).

### 2.4. Scanning Electron Microscopy (SEM)

Microstructural observations were performed using a ZEISS Gemini SEM 300 field-emission SEM (Carl Zeiss Microscopy GmbH, Jena, Germany). Freshly fractured surfaces were gold-coated and examined at 5–15 kV at magnifications of typically 50×, 500×, and 5000×. To provide approximate, image-derived pore-area estimates, representative SEM micrographs of selected samples (GW-M1, GW-Q1, LY-T1, ZG-3) were used to estimate the fraction of the imaged area occupied by visible pores. These values are reported as approximate two-dimensional pore-area estimates from representative micrographs and should be interpreted as indicative rather than definitive measures of three-dimensional pore volume; no calibrated stereological protocol or SEM–EDS was applied.

### 2.5. Firing Temperature Estimation

Firing temperatures were estimated by re-firing thermal expansion for a limited subset of samples (GW-M1, GW-Q1, LY-T1, LY-T2) using a NETZSCH TMA 402F3 (NETZSCH-Gerätebau GmbH, Selb, Germany) thermomechanical analyzer (N_2_ atmosphere, 12 °C·min^−1^, 30–1400 °C). The inflection point in the thermal expansion curve was taken as the estimated original firing temperature [28,29]. Because only four samples were tested, firing temperature results are presented as individual sample observations and should not be generalized to entire site groups.

### 2.6. Bulk Density and Water Absorption

Bulk density and water absorption were determined by the vacuum saturation method using a density balance (FA1204B, Shanghai Jingke Tianmei Scientific Instrument Co., Ltd., Shanghai, China). Density was calculated as ρ = m_1_/(m_2_ − m_3_) × ρ_water_, and water absorption as W_a_ = (m_2_ − m_1_)/m_1_ × 100%. Physical property data were available for 12 of the 21 samples; the remaining 9 samples could not be tested due to insufficient specimen size or conservation restrictions. All physical property comparisons in this paper are limited to these 12 samples; conclusions are not extrapolated to untested specimens.

### 2.7. Compressive Strength

Compressive strength was tested on 40 mm cubic specimens following GB/T 4111–2013 [30] using a servo-hydraulic testing machine. Five parallel specimens per sample were tested; the maximum and minimum were excluded and the mean of the remaining three reported. Compressive strength data are available for 10 of the 21 samples). Because intact standard-format bricks could not be sacrificed from these protected structures, cubes were cut from detached or broken fragments; the standard is therefore applied in an adapted form and the resulting values are treated as comparative indicators rather than code-compliant structural ratings.

### 2.8. Statistical Analysis

All elemental data were z-score standardized prior to multivariate analysis. Because oxide weight-percent data are compositional (constant-sum), and because the absolute oxide totals varied between samples (the residual balance representing unreported volatile and minor components and analytical uncertainty rather than independently measured loss on ignition), all multivariate analyses were additionally repeated on centered log-ratio (CLR) transformed data as a sensitivity test (see Section 3.7 and Supplementary Table S2). The z-score model is retained as the primary presentation to facilitate direct comparison with previous XRF-based brick clustering studies, whereas the CLR model is used as a compositional-data sensitivity test. PCA was performed to reduce dimensionality. K-means clustering (Euclidean distance) was applied with the optimal k determined by the elbow method (WCSS plot, k = 2–6) and silhouette coefficient evaluation. The elbow plot and silhouette scores are presented in Section 3.9 to document the cluster number selection. Both the k = 2 and k = 4 partitions were examined. The k = 2 solution was treated as the primary result, as it has the clearest geochemical interpretation (calcareous versus non-calcareous recipes) and is the most stable across standardizations; the k = 4 solution is reported as a finer-grained, supplementary view that enables comparison with the four-cluster framework of Zhang et al. (2025) [27]. The silhouette coefficients are modest and do not strongly favor any single value of k, consistent with the data forming a near-continuum with one clear weathering outlier rather than a set of sharply separated clusters.

To assess the influence of secondary weathering contamination on multivariate results, two parallel analyses were conducted: (1) full dataset (*n* = 21) and (2) reduced dataset excluding the two most weathered samples, WP-SE2 and WP-SE3 (*n* = 19). Results from both analyses are compared in Section 3.7.

Compositional and physical property differences between Ming (*n* = 4) and Qing (*n* = 4) Great Wall bricks were evaluated using the Mann–Whitney U test (two-tailed, α = 0.05), a non-parametric test appropriate for small sample sizes. Effect sizes were estimated using the rank-biserial correlation coefficient (r_rb_). All analyses were conducted in Python 3.10 using scikit-learn, SciPy v1.17.1, NumPy v2.4.4, and Matplotlib v3.10.9.

## 3. Results

### 3.1. Major-Element Composition

The complete XRF results for all 21 brick samples are presented in Table 2, including nine oxides detected above quantification limits. All samples share a broadly similar silico-aluminous composition (SiO_2_: 39.8–69.3 wt.%; Al_2_O_3_: 12.3–18.5 wt.%), consistent with fired clay products from North China loessial or alluvial deposits. The most notable variation occurs in CaO (1.00–20.24 wt.%), MgO (2.07–6.19 wt.%), and SO_3_ (0–9.82 wt.%). Mean compositions by site group are shown in Table 3.

### 3.2. Physical Properties

Physical property data were obtained for 12 of the 21 samples (Table 4). Among the Great Wall bricks (all 8 samples tested), Ming dynasty specimens exhibit a mean bulk density of 1.65 ± 0.08 g·cm^−3^, mean water absorption of 20.9 ± 2.9%, and mean compressive strength of 8.3 ± 0.4 MPa. The Qing dynasty bricks show higher density (1.79 ± 0.02 g·cm^−3^), lower water absorption (15.9 ± 0.8%), and higher compressive strength (9.0 ± 0.3 MPa). The two Tang Lingyue Temple bricks display the lowest compressive strength in the dataset (5.1 and 6.6 MPa). Two Zhengang Tower bricks (ZG-1 and ZG-3) provided density and water absorption data but no compressive strength, owing to insufficient specimen volume. No physical property data were obtainable for the remaining nine samples (LY-TWD, LY-DXB, ZG-2, ZG-4, ZG-5, WP-SE2–SE4, WP-NE5) due to conservation restrictions on destructive sampling.

### 3.3. SEM Microstructural Observations

**Figure 2:**
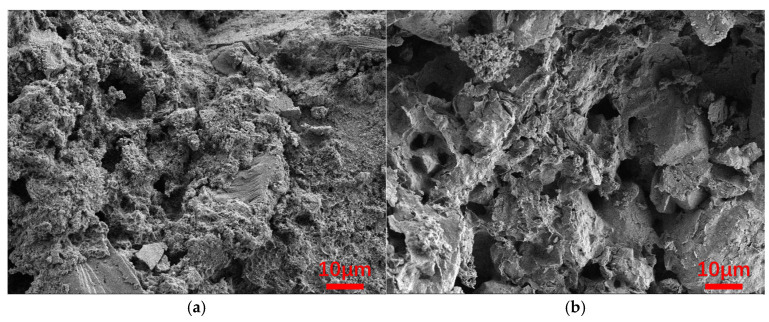

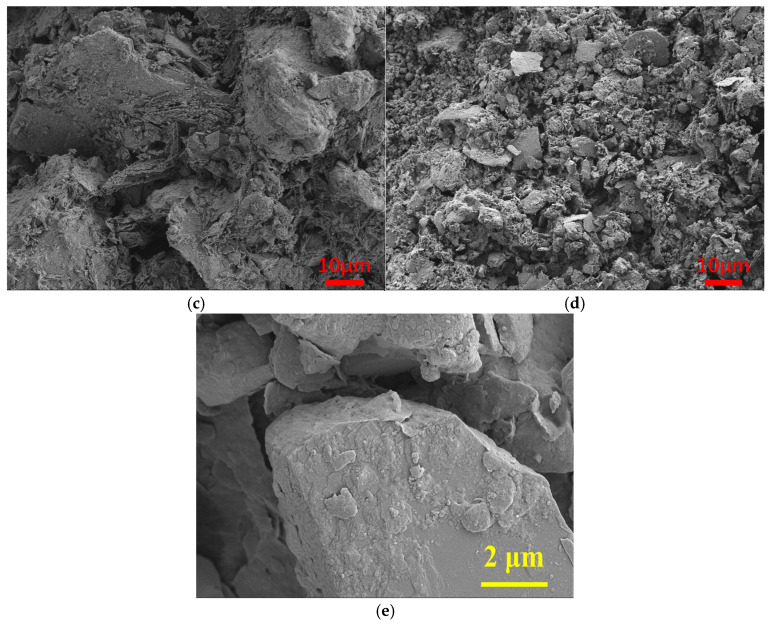
Representative SEM micrographs: (**a**) GW-M1 (Ming Great Wall), porous texture with irregular voids and angular quartz grains; (**b**) GW-Q1 (Qing Great Wall), relatively compact, well-sintered matrix; (**c**) LY-T1 (Lingyue Temple, Tang), open porous microstructure with interconnected voids; (**d**) ZG-3 (Zhengang Tower eave brick), high porosity; (**e**) WP-SE4 (Wanping City), dense matrix showing a partially vitrified groundmass possibly suggestive of localized vitrification or incipient melting. Scale bars: 10 μm in (**a**–**d**), 2 μm in (**e**).

SEM examination shows that the brick samples consist of a fired clay matrix containing irregularly shaped mineral grains with subordinate platy, mica-like particles. Calcite is indicated by XRD in several samples, but SEM morphology alone was not used for phase identification (no SEM–EDS was performed). Representative SEM micrographs for five samples are presented in Figure 2; approximate pore-area estimates were calculated only for four selected samples (GW-M1, GW-Q1, LY-T1, and ZG-3).

Notable inter-group differences are observed. The Qing Great Wall bricks display a relatively compact, well-sintered matrix (qualitatively low pore-area for GW-Q1), consistent with their higher bulk density and lower water absorption. In contrast, the Ming Great Wall brick GW-M1 shows a more porous texture (qualitatively high pore-area), with abundant irregularly shaped voids. The Zhengang Tower eave brick ZG-3 exhibits the highest qualitative pore-area among the four examined samples, consistent with its low density (1.58 g·cm^−3^) and high water absorption (23.3%). The WP-SE4 micrograph shows a locally smooth, partially vitrified interparticle groundmass, possibly reflecting incipient feldspathic melt; because no SEM–EDS or quantitative glass-phase analysis was performed, this interpretation remains tentative. It should be noted that these are qualitative pore-area assessments based on visual inspection of representative 2D micrograph fields, and are indicative rather than definitive.

### 3.4. XRD Mineral Assemblages and Firing Temperature Estimation

**Figure 3:**
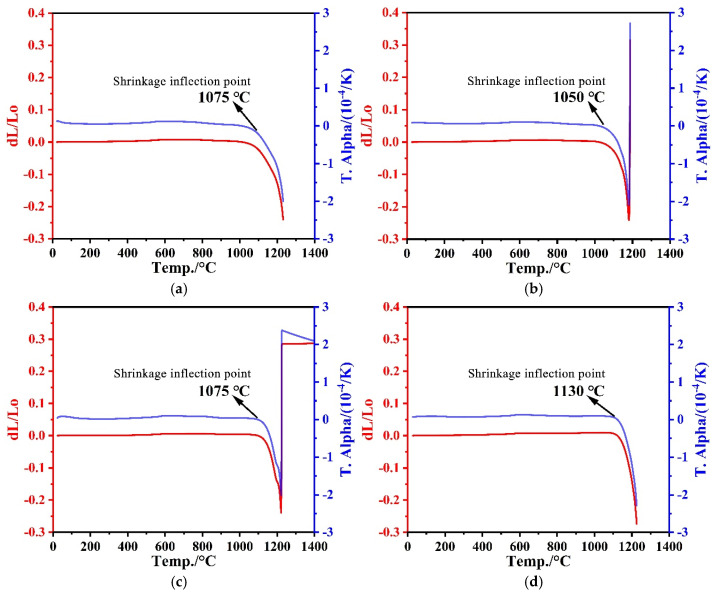
Re-firing thermal expansion curves (dL/L_0_ vs. Temperature) for four selected samples: (**a**) GW − M1, firing temperature ≈ 1075 °C; (**b**) GW − Q1, ≈1050 °C; (**c**) LY − T1, ≈1075 °C; (**d**) LY − T2, ≈1130 °C. Arrows indicate the inflection point where sintering shrinkage resumes, interpreted as the original firing temperature.

XRD analysis identifies two principal mineral assemblage types (Table 5, Figure 1; reference diagnostic reflections are listed in Supplementary Table S3, and sample-level XRD type assignments are summarized in Supplementary Table S6). Type A, observed predominantly in the Ming Great Wall bricks (GW-M1–M3), consists of quartz + anorthite (CaAl_2_Si_2_O_8_) + phengite + calcite. Anorthite is identified by characteristic diffraction peaks at 2θ ≈ 22.0°, 27.8°, and 28.0° (Figure 1a). Type B, observed in the Qing Great Wall, Lingyue, Zhengang, and most Wanping samples, consists of quartz + albite (NaAlSi_3_O_8_) + muscovite/phengite ± calcite ± gypsum, with albite identified by peaks at 2θ ≈ 22.0°, 23.5°, and 27.9° (Figure 1b). The Wanping bricks WP-SE2 and WP-SE3 additionally show gypsum (CaSO_4_·2H_2_O) with a diagnostic peak at 2θ ≈ 11.6° (Figure 1d), interpreted as a secondary weathering product. Re-firing thermal-expansion analysis of the four tested samples yielded estimated original firing temperatures of approximately 1050–1130 °C (Figure 3; Table 5): ≈1075 °C for GW-M1, ≈1050 °C for GW-Q1, and ≈1075 °C and ≈1130 °C for the Tang Lingyue Temple bricks LY-T1 and LY-T2, respectively.

### 3.5. Ming Versus Qing Great Wall Brick Comparison

**Figure 4:**
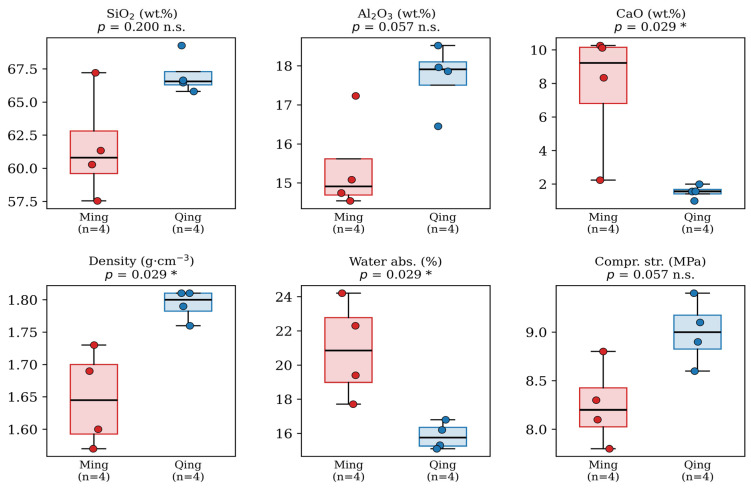
Comparison of Ming (*n* = 4) and Qing (*n* = 4) Great Wall brick compositions and physical properties. Box plots with individual data points; *p*-values from Mann–Whitney U test (* nominal *p* < 0.05, uncorrected; n.s. = not significant).

Table 6 presents Mann–Whitney U test results comparing Ming (*n* = 4) and Qing (*n* = 4) dynasty Great Wall bricks across six compositional and three physical property variables, and Figure 4 shows box plots of six representative variables (SiO_2_, Al_2_O_3_, CaO, density, water absorption, and compressive strength) with individual data points. Despite the small sample sizes, several variables show nominally significant differences at α = 0.05 (CaO: *p* = 0.029; density: *p* = 0.029; water absorption: *p* = 0.029), with large effect sizes (|r_rb_| = 1.0). The SiO_2_ and Al_2_O_3_ differences, while large in magnitude (+5.5 wt.% and +2.3 wt.%, respectively), do not reach nominal significance (*p* = 0.200 and 0.057, respectively), reflecting the within-group variance introduced by the silica-rich, low-CaO Ming outlier GW-M4. Fe_2_O_3_ also reaches nominal significance (*p* = 0.029), but the absolute difference is small (0.28 wt.%) and should be interpreted cautiously given the semi-quantitative nature of the XRF data and the absence of replicate measurements. Because nine variables were tested simultaneously without formal multiple comparison correction, these results should be interpreted as exploratory statistical evidence rather than confirmatory hypothesis tests. Applying Bonferroni correction (α′ = 0.05/9 = 0.0056) would render none of the individual comparisons significant at the corrected threshold, given that the minimum achievable *p*-value with *n* = 4 per group is 0.029. Statistical significance at *n* = 4 per group has limited power, and the *p*-values should be viewed as indicative rather than definitive.

### 3.6. PCA Results

**Figure 5:**
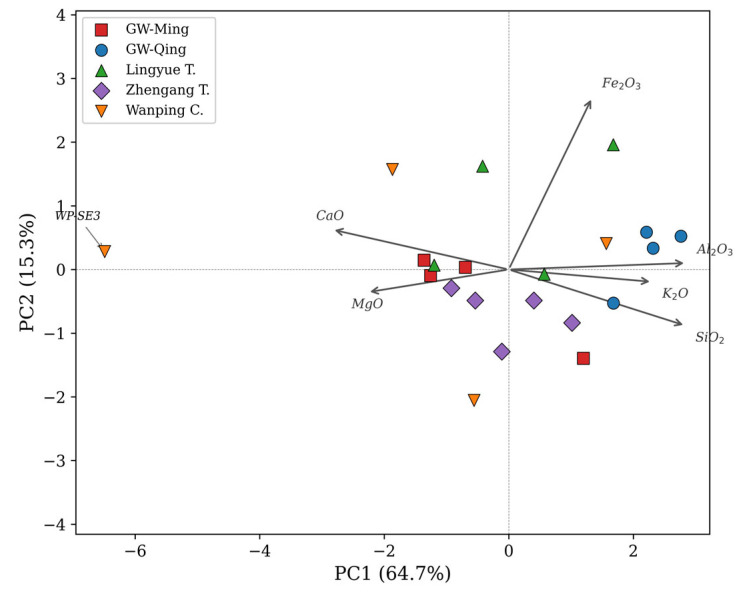
PCA score biplot of six major oxides (*n* = 21). Loading vectors indicate each oxide’s contribution. PC1 (64.7%) separates silico-aluminous from calcareous compositions; PC2 (15.3%) reflects Fe_2_O_3_ variation; the sign of PC2 is arbitrary, and the absolute loading indicates Fe_2_O_3_-dominated variation. WP-SE3 outlier annotated.

Using the primary 6-oxide model, PC1 explains 64.7% and PC2 15.3% of total variance (cumulative 80.1%). PC1 is defined by a contrast between SiO_2_ (+0.470), Al_2_O_3_ (+0.472), and K_2_O (+0.382) on the positive side and CaO (−0.469) and MgO (−0.376) on the negative side. PC2 is dominated by Fe_2_O_3_ (|loading| = 0.919). In the PCA score plot (Figure 5), Qing Great Wall bricks cluster tightly at positive PC1, while Ming bricks occupy the central zone. WP-SE3 is a pronounced outlier at extreme negative PC1 (SiO_2_ = 39.8%, CaO = 20.2%). Removal of WP-SE2 and WP-SE3 from the dataset (*n* = 19 reduced model) reduces the proportion of variance carried by PC1 (PC1: 52.4%, PC2: 20.9%), because the extreme WP-SE3 outlier no longer stretches that axis; the PC1 loading pattern itself is essentially unchanged (cosine similarity of approximately 0.98 with the full-sample PC1), and the silico-aluminous versus calcareous contrast and the overall sample grouping persist, indicating that the grouping pattern is robust to the exclusion of weathered outliers.

### 3.7. Sensitivity Analysis: Extended Element Model and Weathering Outlier Exclusion

An extended 9-oxide PCA (adding Na_2_O, TiO_2_, SO_3_) was performed for comparison. The overall variance structure is similar (PC1: 53.0%, PC2: 17.7%, cumulative 70.7%), with Na_2_O contributing moderately to both PC1 and PC2, and SO_3_ strongly loading on PC2. The Wanping outliers (WP-SE2, WP-SE3) are even more strongly separated in the 9-oxide model, confirming that their anomalous SO_3_ concentrations are the primary driver of their outlier status. Excluding these two samples and repeating both the 6-oxide and 9-oxide PCA/K-means analyses yields groupings that are consistent with the full-dataset results in terms of major cluster membership. This suggests that the core compositional grouping structure is not an artifact of weathering contamination, and supports the robustness of the clustering approach.

**Figure 6:**
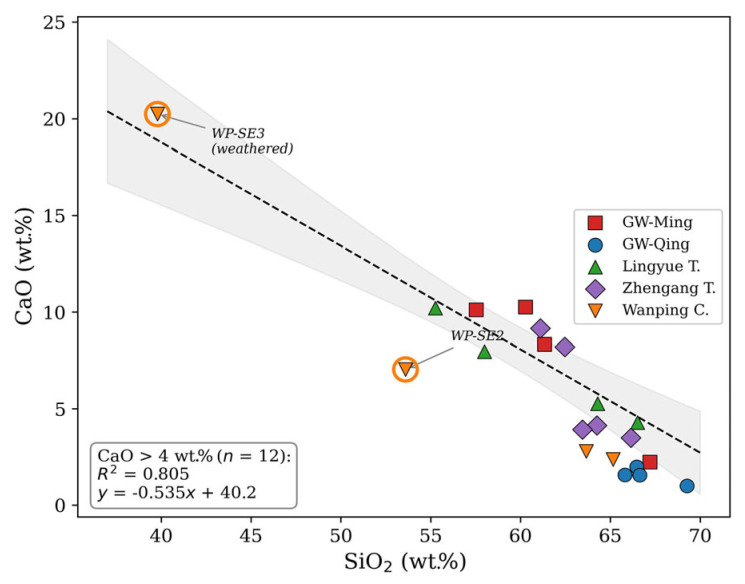
SiO_2_ vs. CaO scatter plot. Dashed line: linear regression for CaO > 4 wt.% (R^2^ = 0.805). Gray shading: 95% confidence band. Circled symbols: weathering-affected samples (WP-SE2, WP-SE3).

As oxide weight-percent data are closed (constant-sum) compositional data, the multivariate analysis was repeated on centered log-ratio (CLR) transformed compositions to confirm that the principal-component structure and the SiO_2_–CaO relationship are not artifacts of closure (Supplementary Table S2). The CLR-PCA reproduced the same silico-aluminous versus calcareous contrast on PC1 (81.6% of variance, versus 64.7% under z-score standardization), the k = 2 partition was identical for all 21 samples under both treatments, and WP-SE3 again formed an isolated single-sample cluster at k = 4. The inverse SiO_2_–CaO correlation was essentially unchanged between the two treatments (Pearson r = −0.918 on raw data; r = −0.920 in CLR coordinates), confirming that it reflects a genuine carbonate-versus-silicate compositional control rather than spurious negative correlation induced by closure. The Pearson correlations reported here were calculated for the full 21-sample compositional space, whereas the regression coefficient in Figure 6 (R^2^ = 0.805) was fitted only to the CaO > 4 wt.% subset (*n* = 12).

### 3.8. SiO_2_–CaO Relationship

A strong inverse linear relationship exists between SiO_2_ and CaO for samples with CaO > 4 wt.% (*n* = 12): CaO = −0.535 × SiO_2_ + 40.2, R^2^ = 0.805 (Figure 6). The anomalous Wanping samples (WP-SE2, WP-SE3) deviate from the regression line, suggesting their elevated CaO partly reflects secondary enrichment rather than primary clay composition. When these two samples are excluded, the regression for CaO > 4 wt.% (*n* = 10) yields R^2^ = 0.70. The inverse trend therefore persists among the unweathered samples; the lower R^2^ relative to the single-source dataset of Zhang et al. (2025; R^2^ = 0.95) [27] is consistent with the inclusion of bricks from more diverse geological sources across four functionally distinct sites. Crucially, this inverse relationship is not an artifact of compositional closure: a centered log-ratio (CLR) transformation yields essentially the same correlation as the raw data (raw r = −0.918; CLR r = −0.920; see Section 3.7).

### 3.9. K-Means Clustering Results

**Figure 7:**
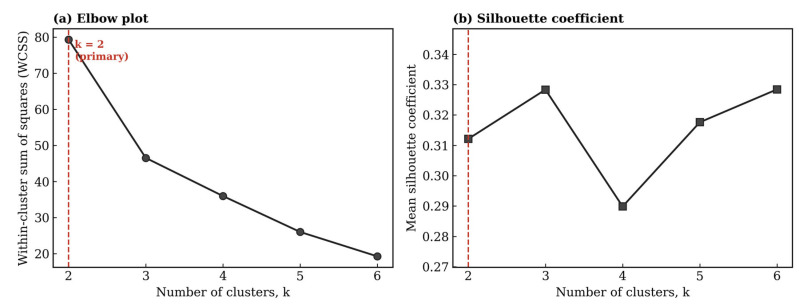
Cluster number selection: (**a**) WCSS elbow plot for k = 2–6; (**b**) mean silhouette coefficient. The coefficients are modest across all k; k = 2 is adopted as the primary partition, with k = 4 reported as a supplementary view (dashed line).

The elbow plot (Figure 7a) shows a progressive decrease in WCSS from k = 2 to k = 6, with the most pronounced inflection at k = 3–4. Silhouette coefficients (Figure 7b) are moderate across all tested values (k = 2: 0.31; k = 3: 0.33; k = 4: 0.29; k = 5: 0.32; k = 6: 0.33), indicating no single k value is strongly preferred by silhouette analysis; all values between 2 and 6 yield similar coefficients (0.29–0.33). We therefore adopt a two-tier approach: k = 2 as the primary clustering, which provides an interpretable first-order calcareous/non-calcareous partition, and k = 4 as a supplementary exploratory analysis that permits finer compositional subtyping and comparison with the four-cluster framework of Zhang et al. (2025) [27]. The k = 4 results should be interpreted as exploratory compositional groupings rather than statistically optimal clusters.

**Figure 8:**
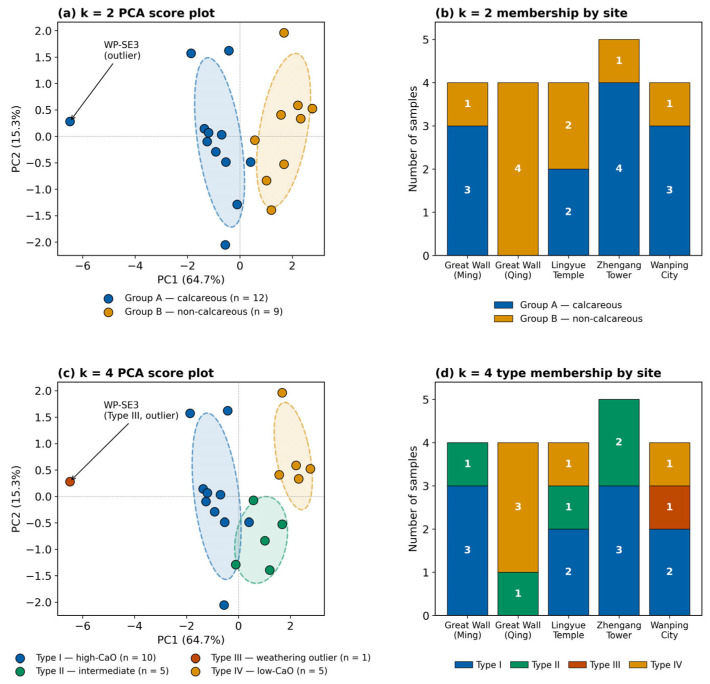
K-means clustering results with dual-tier analysis (k = 2 primary and k = 4 supplementary): (**a**) k = 2 PCA score plot showing Group A (calcareous-dominant, *n* = 12) and Group B (non-calcareous, *n* = 9); (**b**) k = 2 cluster membership by site; (**c**) k = 4 PCA score plot showing four compositional types (Type I = high CaO, Type II = mixed high-SiO_2_/low-to-moderate CaO group, Type III = WP-SE3 weathering outlier, Type IV = low CaO); (**d**) k = 4 compositional type membership by site. Symbols in (**a**,**c**) are colored by cluster/type assignment. Shaded ellipses are 1.5σ covariance ellipses indicating the spread and orientation of each multi-sample cluster, drawn as a visual aid rather than a classification boundary; no ellipse is drawn for the single-sample Type III, and the WP-SE3 weathering outlier (excluded from the Group A ellipse) lies outside the envelopes and is labeled in (**a**,**c**). Numerals on the bars in (**b**,**d**) give sample counts. PC1 and PC2 explain 64.7% and 15.3% of the total variance, respectively.

The primary k = 2 clustering divides the 21 samples into two groups (Figure 8a,b; Table 7): Group A (calcareous-dominant, *n* = 12) encompasses the calcareous Ming Great Wall bricks (GW-M1–M3), the later Lingyue Temple elements (LY-TWD, LY-DXB), most Zhengang Tower bricks (ZG-1, ZG-2, ZG-4, ZG-5), and the three southeast-corner Wanping samples (WP-SE2–SE4), unified by moderate-to-high CaO and MgO (the fourth Wanping sample, WP-NE5, instead falls in Group B). Group B (non-calcareous, *n* = 9) includes GW-M4, all Qing Great Wall bricks, both Tang Lingyue Temple bricks (LY-T1, LY-T2), ZG-3, and WP-NE5, sharing low CaO with high SiO_2_–Al_2_O_3_ contents. This binary partition provides a useful first-order exploratory compositional partition that is largely independent of site provenance.

K-means clustering with k = 4 produces the following compositional types (Figure 8c,d): Type I (*n* = 10) comprises samples characterized by moderate-to-high CaO, including most Ming Great Wall, Lingyue Temple, and Zhengang Tower bricks. Type II (*n* = 5) is a mixed group sharing high SiO_2_ and variable low-to-moderate CaO. Type III (*n* = 1) contains only WP-SE3, the extreme weathering outlier, which is better interpreted as a weathering-altered composition than as a distinct production type. Type IV (*n* = 5) comprises samples with low CaO and high SiO_2_–Al_2_O_3_, including most Qing bricks.

These compositional types show partial, but not exclusive, correspondence to archeological provenance. No cluster is site-exclusive: Type I and Type IV each contain bricks from multiple sites. This result indicates that K-means clustering of XRF data identifies compositional types reflecting raw material chemistry rather than unique site-specific production signatures. The clustering should therefore be interpreted as a tool for compositional classification, not as a direct provenance indicator.

## 4. Discussion

### 4.1. Temporal Differences in Great Wall Brick Composition and Properties

The most notable finding is the compositional and physical property distinction between Ming and Qing Great Wall bricks, supported by nominal, uncorrected Mann–Whitney results for CaO, density, and water absorption (Table 6); because of the small sample size and multiple testing, these comparisons should be treated as exploratory. Most Ming Great Wall bricks, especially GW-M1–M3, are characterized by higher CaO and an anorthite-bearing assemblage (Type A), together with lower density and higher water absorption. GW-M4 is an exception: despite its Ming archeological attribution and the original anorthite-bearing XRD assignment, its low CaO content (2.23 wt.%) and non-calcareous cluster membership indicate that it should be treated as a compositional outlier within the Ming group. This chemistry–mineralogy discrepancy is interpreted cautiously rather than as a mineralogical re-classification. The Qing bricks show low CaO (1.5 ± 0.4 wt.%), albite instead of anorthite (Type B assemblage), and improved physical properties.

This multi-dimensional shift may reflect one or more of the following: (1) a change in the geological source of raw clay from calcareous to non-calcareous deposits; (2) a change in paste preparation or tempering practice; (3) modifications in firing conditions; or (4) some combination thereof. The data do not permit definitive discrimination among these possibilities without additional evidence, such as reference clay samples from historical kiln sites, trace-element fingerprinting, or Sr–Nd isotopic analysis. Nevertheless, the consistency of the compositional and mineralogical shift across all four Qing samples, and the concordant improvement in physical properties, suggest a systematic rather than random change in production inputs or practices between the two dynastic periods.

It should be noted that the within-group variance of the Ming bricks is relatively high (CaO CV = 49%), driven primarily by GW-M4, which has anomalously low CaO (2.23 wt.%) compared with the other three Ming bricks (8.33–10.26 wt.%). Its low CaO is the primary driver of this elevated dispersion, and its inclusion weakens some inter-group statistical comparisons. A sensitivity analysis excluding GW-M4 raises the mean CaO of the remaining three Ming bricks to 9.57 ± 1.08 wt.% (vs. 7.74 ± 3.77 wt.% with GW-M4 included) and yields complete rank separation from the Qing group; however, with *n* = 3 vs. 4, the exact two-tailed Mann–Whitney *p*-value is 0.057, so this sensitivity result is treated descriptively rather than inferentially. Its anomalously low CaO (2.23 wt.%) coexists with the anorthite reported for this sample by the original laboratory XRD analysis, and a re-examined diffractogram (Supplementary Figure S2) confirms this anorthite-bearing (Type A) assemblage—quartz, anorthite, phengite, and subordinate calcite. We therefore retain the original Type A assignment and interpret the low bulk CaO as a genuine compositional feature, qualitatively consistent with the weak calcite reflections of an otherwise anorthite-bearing brick, rather than an analytical artifact or a basis for mineralogical re-classification; GW-M4 is best regarded as an anorthite-bearing but CaO-poor compositional outlier within the Ming group. We retain all samples in the primary analysis to avoid selective data exclusion, but the sensitivity result reinforces the robustness of the Ming–Qing compositional distinction when GW-M4 is treated as a possible outlier.

### 4.2. Lingyue Temple Tang Dynasty Bricks: Low Mechanical Performance

The Tang Lingyue Temple bricks (LY-T1, LY-T2) exhibit the lowest compressive strength in the dataset (5.1 and 6.6 MPa), despite individual firing temperature estimates of 1075 and 1130 °C, respectively. The combination of moderately high CaO (4.3–5.3 wt.%) and visible pore development (qualitatively moderate pore-area for LY-T1) is consistent with, but does not prove, a contribution from carbonate decomposition and incomplete sintering despite adequate firing temperatures. [31] This observation underscores that firing temperature alone is insufficient to predict mechanical performance without considering clay body chemistry and resultant pore structure.

The occurrence of calcite in several samples with estimated firing temperatures above 1000 °C should be interpreted with caution. Calcite may represent residual carbonate protected within coarse grains, secondary recarbonation after firing, contamination from lime-bearing mortars or conservation deposits, or later weathering products, rather than a purely primary firing phase. For this reason, calcite is not used in isolation as a firing-temperature indicator in this study; instead, the temperature interpretation relies on the combined evidence of feldspar assemblages (the anorthite-to-albite transition), thermal-expansion behavior, and bulk CaO contents.

### 4.3. Wanping City Brick Heterogeneity and Weathering Effects

The Wanping City bricks exhibit the highest compositional variability (CaO CV = 103%). WP-SE3 is a clear outlier with extremely high CaO (20.24 wt.%), SO_3_ (9.82%), and Cl (2.10%), consistent with severe sulfate and chloride weathering [14,26]. WP-SE2 is also strongly sulfate-enriched (SO_3_ 7.21 wt.%) but, unlike WP-SE3, carries only minor chloride (Cl 0.43 wt.%). The sensitivity analysis (Section 3.7) confirms that excluding these two samples does not alter the core PCA/clustering structure and does not increase the R^2^ of the SiO_2_–CaO regression (R^2^ = 0.805 for the CaO > 4 wt.% subset of 12 samples; excluding WP-SE2 and WP-SE3 lowers it to R^2^ = 0.704), although the inverse trend persists clearly among the less weathered samples. This reinforces the importance of weathering assessment before interpreting XRF data as an indicator of raw material source. The MgO contents of the less weathered Wanping samples remain variable (WP-SE4 = 6.16 wt.%, WP-NE5 = 3.71 wt.%), so a distinct Mg-rich clay source is possible but cannot be confirmed from this small subset. Beyond the Wanping samples, the later Lingyue Temple element LY-DXB also shows a moderate chloride enrichment (Cl 1.67 wt.%) but without associated sulfate (SO_3_ not detected), pointing to localized secondary chloride-salt contamination rather than the combined sulfate–chloride attack recorded in WP-SE3. Because the major-oxide composition of LY-DXB (SiO_2_, Al_2_O_3_, CaO) remains within the range of the other calcareous bricks, this chloride does not make it a compositional outlier, and it retains its calcareous (k = 2 Group A)/Type I (k = 4) assignment; the chloride is therefore interpreted as a secondary weathering signal rather than a primary clay feature, consistent with the cautious treatment of secondary salts elsewhere in this study.

### 4.4. SiO_2_–CaO Geochemical Constraint

The inverse SiO_2_–CaO relationship (R^2^ = 0.805 for the CaO > 4 wt.% subset, *n* = 12; the full-sample Pearson correlation is reproduced under a centered log-ratio transformation, confirming it is not a closure artifact) is consistent with the pattern reported by Zhang et al. (2025, R^2^ = 0.95) [27] for a separate Yanqing–Fengtai dataset. The lower R^2^ likely reflects the inclusion of bricks from more diverse geological sources and the influence of secondary weathering. These data are consistent with the interpretation that Beijing-area brick clays share a common sedimentary geochemical signature, with CaO/SiO_2_ ratios varying primarily due to differences in carbonate mineral content within the raw clay [32].

### 4.5. Interpretation of K-Means Compositional Types

The K-means clustering identifies four compositional types that show partial but not exclusive correspondence to archeological provenance. This result is consistent with the expectation that XRF-based clustering reflects raw material chemistry rather than site-specific production practices, because different sites could have used clays from similar or overlapping geological sources. The single-sample Type III (WP-SE3) should not be interpreted as a distinct production type but rather as a weathering-altered end-member. Future studies with larger sample sizes may benefit from additional clustering validation approaches, such as fuzzy C-means or Gaussian mixture models, and from the full dual-channel XRD–XRF cross-matrix approach of Zhang et al. (2025) [27].

### 4.6. Preliminary Implications for Heritage Conservation

The compositional, mineralogical, and physical property differences documented in this study provide preliminary guidance for material-compatible restoration. For the tested Miyun Great Wall samples, these data suggest that replacement-brick screening should consider provisional dynastic reference ranges: more calcareous compositions for most Ming samples and non-calcareous compositions for Qing samples. However, these recommendations should be considered provisional until validated by larger sample sizes and supplemented by additional durability indicators such as freeze–thaw resistance, salt crystallization resistance, capillary water absorption coefficients, and thermal expansion compatibility [6,33,34,35]. For Wanping City, the active sulfate and chloride weathering mechanisms must be addressed (e.g., through desalination treatment) before replacement bricks are installed. For the Tang Lingyue Temple bricks, the very low compressive strength suggests that structural reinforcement may be required in addition to material replacement.

### 4.7. Limitations

Several limitations constrain the interpretation of these results. (1) Sample sizes per site group are small (2–5 for non-Great-Wall sites), limiting statistical power and precluding robust inter-group significance testing for these groups. All conclusions regarding site-level differences should be interpreted as preliminary indications rather than definitive findings. (2) Physical property data were obtainable for only 12 of 21 samples (57%), and compressive strength for only 10 samples. Comparisons involving physical properties are restricted to tested specimens and are not extrapolated to untested samples. (3) Some samples from Zhengang Tower and later Lingyue Temple elements lack secure chronological attribution. (4) The absence of reference clay/soil samples precludes direct clay-to-brick matching. (5) Only four samples were tested for firing temperature; generalizations about site-level or period-level firing regimes are not supported. (6) This study applies K-means clustering to XRF elemental data only and does not implement the full dual-channel XRD pattern clustering methodology of Zhang et al. (2025) [27]. (7) Qualitative SEM pore-area assessments are based on limited 2D image analysis and serve as indicative observations.

## 5. Conclusions

The integrated application of WD-XRF, XRD, SEM (with qualitative pore-area assessment), thermal expansion analysis, physical property testing, and K-means clustering to 21 blue brick samples from four Beijing-area sites yields findings that can be organized into three categories of confidence.

### 5.1. Main Descriptive Findings

(1) Among the tested Great Wall bricks, Ming and Qing dynasty specimens differ in CaO content (nominal *p* = 0.029, uncorrected), with a concurrent shift from anorthite-bearing (Type A) to albite-bearing (Type B) mineral assemblages. Qing bricks also show higher density and lower water absorption in the tested subset (nominal *p* = 0.029 for both, uncorrected). (2) Two Wanping City bricks (WP-SE2, WP-SE3) are sulfate-enriched, and WP-SE3 additionally shows elevated chloride and represents the severe sulfate–chloride weathering end-member; this enrichment substantially distorts their primary compositions. Sensitivity analysis confirms that exclusion of these outliers does not alter the core compositional grouping structure. (3) A strong inverse SiO_2_–CaO relationship (R^2^ = 0.805 for the CaO > 4 wt.% subset, *n* = 12) provides preliminary support for a regional geochemical tendency reported by Zhang et al. (2025) [27].

### 5.2. Interpretive Implications

(1) The Ming–Qing compositional and physical property differences suggest a systematic change in raw material sourcing, paste preparation, or production practice, though the specific mechanism cannot be determined from the present data alone. (2) K-means clustering (k = 4) groups bricks into compositional types that partially correspond to site provenance, but the clustering should be interpreted as reflecting raw material chemistry rather than unique site-specific production signatures. (3) The Tang Lingyue Temple bricks exhibit the lowest compressive strength in the dataset despite moderate-to-high firing temperatures, indicating that firing temperature alone does not explain mechanical performance and that clay-body chemistry and pore structure likely contributed to the low strength.

### 5.3. Limitations and Future Directions

The conclusions of this study are constrained by small sample sizes (2–5 per non-Great-Wall group), incomplete physical property data (12/21 samples), limited firing temperature measurements (4/21 samples), and the absence of reference clay samples. Future work should: (1) expand sample sizes where conservation permits; (2) integrate trace-element and Sr–Nd isotopic analyses for more robust provenance discrimination; (3) complete physical property characterization; (4) apply XRD full-diffractogram clustering to implement the complete dual-channel methodology of Zhang et al. (2025) [27]; (5) collect reference clay samples from historically documented kiln sites; and (6) conduct durability testing (freeze–thaw, salt crystallization, capillary absorption) to strengthen conservation recommendations.

## Figures and Tables

**Table 1:** Summary of blue brick samples.

Site	Code	Period	*n*	Dimensions (cm)
Great Wall, Miyun	GW-M1–M4	Ming	4	~44.4 × 21.9 × 9.6
Great Wall, Miyun	GW-Q1–Q4	Qing	4	~36.3 × 17.7 × 8.8
Lingyue Temple	LY-T1, T2	Tang	2	38.6 × 19.5 × 6.7 *
Lingyue Temple	LY-TWD, DXB	Uncertain	2	—
Zhengang Tower	ZG-1 to ZG-5	Uncertain	5	—
Wanping City	WP-SE2–SE4, NE5	Ming	4	—
Total			21	

* Dimension available for LY-T2 only. ‘Uncertain’ denotes samples lacking secure chronological attribution.

**Table 2:** XRF major oxide compositions (wt.%) of all 21 blue brick samples.

Sample	SiO_2_	Al_2_O_3_	Fe_2_O_3_	CaO	K_2_O	MgO	Na_2_O	TiO_2_	SO_3_
GW-M1	60.28	14.54	5	10.26	2.44	4.12	2.29	0.62	n.d.
GW-M2	61.34	15.08	5.05	8.33	2.36	3.54	2.16	n.d.	n.d.
GW-M3	57.54	14.74	5.05	10.12	2.52	4.59	2.52	n.d.	0.89
GW-M4	67.21	17.23	4.78	2.23	2.43	2.68	2.13	0.78	n.d.
GW-Q1	66.47	17.86	5.27	1.99	2.77	2.63	1.89	0.79	n.d.
GW-Q2	66.64	17.96	5.34	1.56	2.62	2.75	1.87	0.74	n.d.
GW-Q3	69.27	16.45	5.06	1	2.48	2.25	2.21	0.81	n.d.
GW-Q4	65.81	18.52	5.32	1.57	2.88	2.52	2.15	0.8	n.d.
LY-T1	64.3	16.62	5.65	5.27	2.71	2.56	1.65	0.68	n.d.
LY-T2	66.51	15.55	5.09	4.29	2.24	2.07	3.04	0.68	n.d.
LY-TWD	55.26	14.15	4.99	10.21	2.72	3.65	3.76	n.d.	2.98
LY-DXB	57.99	15.06	5.45	7.97	2.42	3.36	4.6	n.d.	n.d.
ZG-1	62.47	14.99	4.93	8.2	2.48	3.48	1.93	0.73	n.d.
ZG-2	64.25	15.39	4.8	4.13	2.5	4.06	1.96	0.84	n.d.
ZG-3	66.15	15.94	4.94	3.49	2.7	3	2.08	0.7	n.d.
ZG-4	61.1	14.55	4.98	9.16	2.56	4.14	1.85	0.7	n.d.
ZG-5	63.45	15.08	5.07	3.92	2.95	4.87	2.19	n.d.	0.63
WP-SE2	53.6	14.17	5.47	7.04	2.36	6.19	2.25	0.82	7.21
WP-SE3	39.79	12.27	4.72	20.24	1.69	6.05	2.4	0.55	9.82
WP-SE4	63.66	15.22	4.67	2.79	2.68	6.16	2.11	0.74	1.21
WP-NE5	65.15	17.08	5.3	2.37	2.73	3.71	2.03	0.7	0.35

n.d. = not detected or below the quantification limit. Cl was detected in LY-DXB (1.67%), WP-SE2 (0.43%), and WP-SE3 (2.10%), and was below the quantification limit in all other samples; the complete element dataset, including Cl and P_2_O_5_, is provided in Supplementary Table S4. The elevated Cl in these samples reflects secondary weathering and should be interpreted cautiously.

**Table 3:** Mean major oxide compositions (wt.%, mean ± SD) by site group.

Site Group	*n*	SiO_2_	Al_2_O_3_	Fe_2_O_3_	CaO	K_2_O	MgO
GW (Ming)	4	61.6 ± 4.1	15.4 ± 1.2	5.0 ± 0.1	7.7 ± 3.8	2.4 ± 0.1	3.7 ± 0.8
GW (Qing)	4	67.1 ± 1.5	17.7 ± 0.9	5.2 ± 0.1	1.5 ± 0.4	2.7 ± 0.2	2.5 ± 0.2
Lingyue T.	4	61.0 ± 5.3	15.3 ± 1.0	5.3 ± 0.3	6.9 ± 2.7	2.5 ± 0.2	2.9 ± 0.7
Zhengang T.	5	63.5 ± 1.9	15.2 ± 0.5	4.9 ± 0.1	5.8 ± 2.7	2.6 ± 0.2	3.9 ± 0.7
Wanping C.	4	55.6 ± 11.7	14.7 ± 2.0	5.0 ± 0.4	8.1 ± 8.4	2.4 ± 0.5	5.5 ± 1.2

**Table 4:** Physical properties of tested blue brick samples (12 of 21).

Sample	Density (g·cm^−3^)	Water Abs. (%)	Compressive Strength (MPa)	SEM Pore-Area (Qualitative)
GW-M1	1.57	24.2	8.1	High
GW-M2	1.6	22.3	8.8	—
GW-M3	1.69	19.4	8.3	—
GW-M4	1.73	17.7	7.8	—
GW-Q1	1.81	15.3	9.4	Low
GW-Q2	1.81	15.1	9.1	—
GW-Q3	1.76	16.8	8.6	—
GW-Q4	1.79	16.2	8.9	—
LY-T1	1.67	19.2	5.1	Moderate
LY-T2	1.66	20.7	6.6	—
ZG-1	1.74	17.1	—	—
ZG-3	1.58	23.3	—	High

— = data not available. SEM pore-area (qualitative): relative visual assessment (low/moderate/high) of pore-area from representative SEM micrographs, not a calibrated porosity measurement.

**Table 5:** XRD mineral assemblages and individual firing temperature estimates.

Sample Group	Mineral Assemblage	Type	Firing Temp. (Tested Sample)
GW-M1–M3 (*n* = 3)	Qz + An + Phg + Cal	A	1075 ± 20 °C (GW-M1 only)
GW-M4 (*n* = 1)	Qz + An + Phg + Cal	A †	Not tested
GW Qing (*n* = 4)	Qz + Ab + Phg + Cal	B	1050 ± 20 °C (GW-Q1 only)
LY Tang (*n* = 2)	Qz + Ab + Phg + Cal	B	1075/1130 ± 20 °C (LY-T1/T2)
LY-TWD, DXB	Qz + Ab + Phg + Cal	B	Not tested
ZG-1 to ZG-5	Qz + Ab + Phg + Cal	B	Not tested
WP-SE4, NE5	Qz + Ab + Ms	B	Not tested
WP-SE2	Qz + Ab + Ms + Gyp	B*	Not tested
WP-SE3	Qz + Ab + Ms + Gyp + Cal	B*	Not tested

B* = Type B assemblage with secondary gypsum. † GW-M4 shows the anorthite-bearing (Type A) assemblage reported by the original XRD analysis but is a compositional outlier (CaO 2.23 wt.%) that groups with the non-calcareous cluster (Section 3.9).

**Table 6:** Mann–Whitney U test comparing Ming and Qing Great Wall bricks.

Variable	Ming Mean	Qing Mean	Δ (Qing − Ming)	U (Ming)	*p*-Value	r_rb_
SiO_2_ (wt.%)	61.6	67.1	5.5	3	0.2	−0.625
Al_2_O_3_ (wt.%)	15.4	17.7	2.3	1	0.057	−0.875
Fe_2_O_3_ (wt.%)	4.97	5.25	0.28	0	0.029 *	−1.00
CaO (wt.%)	7.74	1.53	−6.2	16	0.029 *	1
K_2_O (wt.%)	2.44	2.69	0.25	1	0.057	−0.88
MgO (wt.%)	3.73	2.54	−1.2	15	0.057	0.88
Density (g·cm^−3^)	1.65	1.79	0.14	0	0.029 *	−1.00
Water abs. (%)	20.9	15.9	−5.0	16	0.029 *	1
Compressive strength (MPa)	8.3	9	0.7	1	0.057	−0.875

* Nominally significant at α = 0.05 (uncorrected). r_rb_ = 2U_Ming/(*n*_1_ × *n*_2_) − 1, with U computed for the Ming group; positive r_rb_ indicates higher values in the Ming group, negative r_rb_ indicates higher values in the Qing group. With *n* = 4 per group, the minimum achievable *p*-value is 0.029; none survive Bonferroni correction (α′ = 0.05/9 = 0.0056).

**Table 7:** K-means cluster/group assignments for all 21 samples.

Sample	CaO (wt.%)	XRD Type	k = 2 Group	k = 4 Type	Weathering
GW-M1	10.26	A	A (calcareous)	I	—
GW-M2	8.33	A	A (calcareous)	I	—
GW-M3	10.12	A	A (calcareous)	I	—
GW-M4	2.23	A ^†^	B (non-calcareous)	II	—
GW-Q1	1.99	B	B (non-calcareous)	IV	—
GW-Q2	1.56	B	B (non-calcareous)	IV	—
GW-Q3	1	B	B (non-calcareous)	II	—
GW-Q4	1.57	B	B (non-calcareous)	IV	—
LY-T1	5.27	B	B (non-calcareous)	IV	—
LY-T2	4.29	B	B (non-calcareous)	II	—
LY-TWD	10.21	B	A (calcareous)	I	—
LY-DXB	7.97	B	A (calcareous)	I	—
ZG-1	8.2	B	A (calcareous)	I	—
ZG-2	4.13	B	A (calcareous)	II	—
ZG-3	3.49	B	B (non-calcareous)	II	—
ZG-4	9.16	B	A (calcareous)	I	—
ZG-5	3.92	B	A (calcareous)	I	—
WP-SE2	7.04	B	A (calcareous)	I	Sulfate
WP-SE3	20.24	B*	A (calcareous)	III (outlier)	Severe
WP-SE4	2.79	B	A (calcareous)	I	—
WP-NE5	2.37	B	B (non-calcareous)	IV	—

XRD type: A = anorthite-bearing; B = albite-bearing; B* = albite-bearing with secondary gypsum. The XRD type denotes the dominant feldspar species (Ca-rich anorthite vs. Na-rich albite) and does not by itself indicate bulk CaO content; several albite-bearing (Type B) samples retain calcite and have high bulk CaO. The k = 2 group denotes the two-cluster K-means partition computed on six z-scored oxides and is not a CaO threshold; a few low-CaO samples with elevated MgO and Fe_2_O_3_ (e.g., WP-SE4) therefore fall in the calcareous-dominant cluster. k = 4 Type III = weathering outlier (not a production type). ^†^ GW-M4’s original laboratory XRD analysis assigned an anorthite-bearing (Type A) assemblage; however, its bulk CaO (2.23 wt.%) is anomalously low for an anorthite-rich brick and it groups with the non-calcareous cluster. We retain the original Type A XRD assignment and treat GW-M4 as a compositional outlier, interpreting this chemistry–mineralogy discrepancy cautiously. Weathering: severe = SO_3_ > 7%, Cl > 2%; sulfate = SO_3_ > 7%.

## Data Availability

The original contributions presented in this study are included in the article/Supplementary Materials. Further inquiries can be directed to the corresponding author.

## Supplementary Materials

The following supporting information can be downloaded at: https://www.mdpi.com/article/10.3390/ma19122666/s1, Figure S1: Stacked experimental XRD patterns of representative Tang, Ming, and Qing blue bricks (LY-T1, GW-M1, and GW-Q1) overlaid with reference powder-diffraction (PDF) stick patterns; Figure S2: Re-examined experimental X-ray diffractogram of the Ming Great Wall outlier GW-M4, annotated with its identified mineral phases; Table S1: Sample-by-sample archaeological and sampling context, with the basis and confidence of each chronological attribution; Table S2: Sensitivity of the multivariate results to data treatment: z-score standardization versus centred log-ratio (CLR) transformation; Table S3: Reference diagnostic reflections used for XRD phase identification and for assigning the Type A/Type B/Type B* assemblages; Table S4: Complete WD-XRF major- and minor-element compositions (wt.%) for all 21 samples, including P_2_O_5_, Cl, and the unanalysed balance (“Other”); Table S5: Principal component loadings (z-score-standardized PCA on six oxides) and variance explained; Table S6: K-means cluster assignments per sample (k = 2 and k = 4) together with the XRD assemblage type.

## Abbreviations

The following abbreviations are used in this manuscript:

CLR	Centered log-ratio (transformation)
PCA	Principal component analysis
XRD	X-ray diffraction
XRF	X-ray fluorescence
