# Supplementary material for: Multi-Technique Characterization of Historic Blue Bricks from Beijing: Compositional Grouping, Weathering Assessment, and Conservation Implications
Source: Materials (Basel). 2026 Jun 21;19(12):2666. doi: 10.3390/ma19122666 (PMC13302883; doi:10.3390/ma19122666)
Supplement: Supplementary file 1 [file materials-19-02666-s001.zip › Supplementary Materials materials-4365387.pdf]

# Supplementary Materials

**Figure S1.** Stacked experimental XRD patterns of representative Tang, Ming, and Qing blue bricks (corresponding to LY-T1, GW-M1, and GW-Q1 in Figure 1, panels c, a, and b), shown with the reference powder-diffraction (PDF) stick patterns used for phase identification.

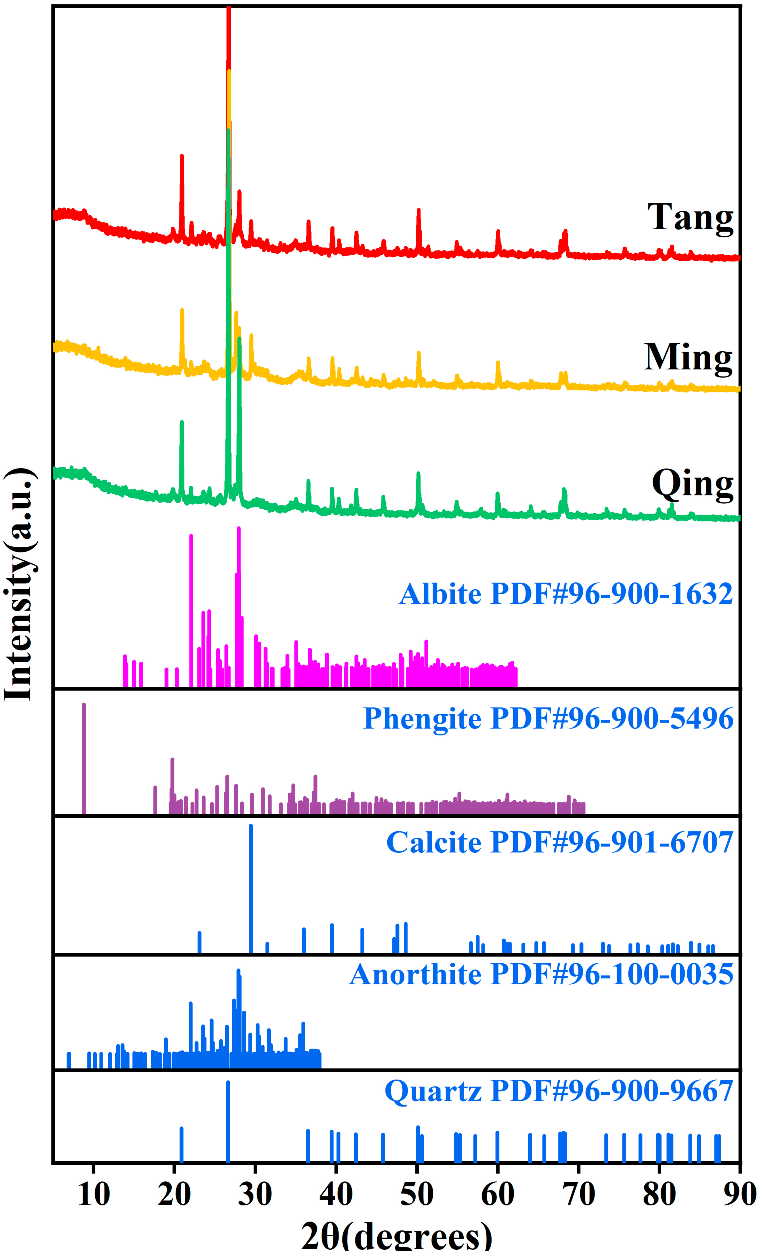


**Figure S1.** Experimental XRD patterns (Tang, red; Ming, gold; Qing, green) overlaid with reference PDF cards: Albite (PDF#96-900-1632), Phengite (PDF#96-900-5496), Calcite (PDF#96-901-6707), Anorthite (PDF#96-100-0035), and Quartz (PDF#96-900-9667). The three experimental patterns are visually similar but are different raw data files; the distinction between the anorthite-bearing (Type A) and albite-bearing (Type B) assemblages is made against the reference patterns shown. Gypsum reference sticks are not overlaid; gypsum was identified from its diagnostic 11.6° reflection as summarized in Table S3.

**Figure S2.** Re-examined experimental X-ray diffractogram of the Ming Great Wall outlier GW-M4, annotated with its identified mineral phases.

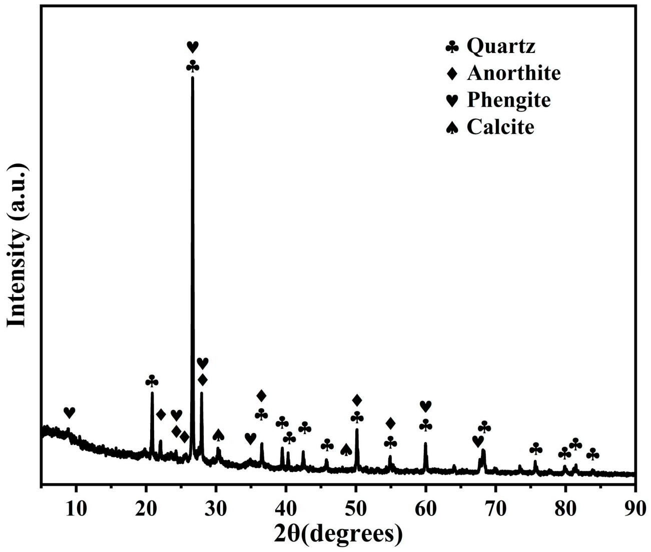


**Figure S2.** Experimental X-ray diffractogram of sample GW-M4 (Ming dynasty, Miyun Great Wall), annotated with the identified phases quartz, anorthite, phengite, and calcite. The presence of anorthite confirms the anorthite-bearing (Type A) assemblage originally reported for this sample, while the relatively weak calcite reflections are qualitatively compatible with its low bulk CaO content (2.23 wt.%); GW-M4 is therefore interpreted as an anorthite-bearing but CaO-poor compositional outlier within the Ming group (see main text, Section 4.1).

**Table S1.** Sample-by-sample archaeological and sampling context, with the basis and confidence of each chronological attribution.

| Sample | Site | Architectural element | Sampling context | Dating basis | Attribution confidence |
|---|---|---|---|---|---|
| GW-M1 | Great Wall (Miyun) | Watchtower masonry | Detached/broken fragment | Typological (Ming) | Probable |
| GW-M2 | Great Wall (Miyun) | Watchtower masonry | Detached/broken fragment | Typological (Ming) | Probable |
| GW-M3 | Great Wall (Miyun) | Watchtower masonry | Detached/broken fragment | Typological (Ming) | Probable |
| GW-M4 | Great Wall (Miyun) | Watchtower masonry | Detached/broken fragment | Typological (Ming) | Probable (mineralogical/compositional outlier; see main text) |
| GW-Q1 | Great Wall (Miyun) | Watchtower masonry | Detached/broken fragment | Typological (Qing) | Probable |
| GW-Q2 | Great Wall (Miyun) | Watchtower masonry | Detached/broken fragment | Typological (Qing) | Probable |
| GW-Q3 | Great Wall (Miyun) | Watchtower masonry | Detached/broken fragment | Typological (Qing) | Probable |
| GW-Q4 | Great Wall (Miyun) | Watchtower masonry | Detached/broken fragment | Typological (Qing) | Probable |

| | | | | | |
|---|---|---|---|---|---|
| LY-T1 | Lingyue Temple | Temple masonry | Detached/broken fragment | Thermoluminescence (TL): 1210 ± 120 yr BP; report no. BJHZ1138 | Secure (absolute TL dating) |
| LY-T2 | Lingyue Temple | Temple masonry | Detached/broken fragment | Typological (Tang) | Probable |
| LY-TWD | Lingyue Temple | West wall, Tianwang Hall | Detached/broken fragment | Typological (later element) | Uncertain |
| LY-DXB | Lingyue Temple | South sill wall, Mahavira Hall | Detached/broken fragment | Typological (later element) | Uncertain |
| ZG-1 | Zhengang Tower | Buddhist niche structure | Detached/broken fragment | Typological | Uncertain |
| ZG-2 | Zhengang Tower | Buddhist niche structure | Detached/broken fragment | Typological | Uncertain |
| ZG-3 | Zhengang Tower | Buddhist niche structure (eave brick) | Detached/broken fragment | Typological | Uncertain |
| ZG-4 | Zhengang Tower | Buddhist niche structure | Detached/broken fragment | Typological | Uncertain |
| ZG-5 | Zhengang Tower | Buddhist niche structure | Detached/broken fragment | Typological | Uncertain |
| WP-SE2 | Wanping City | Southeast corner tower | Detached/broken fragment | Typological (late Ming) | Probable (heavily weathered) |
| WP-SE3 | Wanping City | Southeast corner tower | Detached/broken fragment | Typological (late Ming) | Probable (heavily weathered; compositional outlier) |
| WP-SE4 | Wanping City | Southeast corner tower | Detached/broken fragment | Typological (late Ming) | Probable |
| WP-NE5 | Wanping City | Northeast corner tower | Detached/broken fragment | Typological (late Ming) | Probable |

**Note:** Attribution-confidence categories: Secure = supported by absolute dating (thermoluminescence); Probable = typological attribution with documented architectural provenance; Uncertain = typological attribution only, lacking secure chronological control (as noted in the main text for the Zhengang Tower bricks and the later Lingyue Temple elements LY-TWD and LY-DXB). All specimens were detached or broken fragments collected during conservation work. Possible later repair/replacement was not separately assessed. The LY-T1 thermoluminescence date (1210 ± 120 yr BP, consistent with the Tang attribution) was determined by Beijing Hanzhen Ceramics Technology Co., Ltd. (report no. BJHZ1138).

**Table S2.** Sensitivity of the multivariate results to the data treatment: z-score standardization (primary) versus centred log-ratio (CLR) transformation followed by standardization.

| Feature | z-score (primary) | CLR + standardization |
|---|---|---|
| Variance on PC1 (silico-aluminous vs. calcareous contrast) | 64.7% | 81.6% |
| k = 2 partition (calcareous vs. non-calcareous) | — | Identical membership for all 21 samples |
| WP-SE3 behaviour at k = 4 | Isolated single-sample cluster | Isolated single-sample cluster |
| $SiO_2$–CaO correlation (full 21 samples) | Pearson r = −0.918 | Pearson r = −0.920 |

**Note:** CLR addresses the constant-sum (closure) property of oxide weight-percent data. Because the principal contrast, the two-group partition, and the $SiO_2$–CaO relationship are all reproduced under CLR, the z-score results reported in the main text are not artefacts of compositional closure.

**Table S3.** Reference diagnostic reflections used for XRD phase identification and for assigning the Type A / Type B / Type B* assemblages. Group-level assemblages per sample set are given in Table 5 of the main text; experimental patterns against these references are shown in Figure S1.

| Mineral phase. | Reference card (PDF#) | Characteristic reflections (reference 2θ, °) | Diagnostic role |
|---|---|---|---|
| Quartz | 96-900-9667 | 20.9, 26.6 (strongest), 36.5, 50.1, 59.9, 68.1 | Ubiquitous (all samples) |
| Anorthite ($CaAl_2Si_2O_8$) | 96-100-0035 | 22.0, 24.0, 27.8–28.1 (characteristic multiplet) | Defines the anorthite-bearing Type A assemblage |
| Albite ($NaAlSi_3O_8$) | 96-900-1632 | 22.0, 23.5, 27.7–28.0 (strongest near 28°) | Defines the albite-bearing Type B assemblage |
| Phengite / muscovite | 96-900-5496 | 8.8 (002, low-angle), 17.7, 19.8, 26.8 | Residual mica; present in Type A and Type B |
| Calcite ($CaCO_3$) | 96-901-6707 | 23.0, 29.4 (strongest, 104), 36.0, 39.4, 43.2, 47.5 | Carbonate; interpreted cautiously (Section 4.2) |
| Gypsum ($CaSO_4{\cdot}2H_2O$) | — | 11.6 (020, diagnostic), 20.7, 29.1, 31.1 | Secondary weathering phase; defines Type B* (WP-SE2 and WP-SE3) |

**Note:** XRD analysis is qualitative; values are reference (PDF) positions subject to minor sample-dependent shifts. The Type A vs. Type B distinction is based on the dominant feldspar species (anorthite vs. albite) and does not by itself indicate bulk CaO content (see main text, Table 7 footnote). Gypsum was identified from its diagnostic 11.6° (020) reflection; because no gypsum PDF stick pattern was overlaid, its reference card is not assigned (—).

**Table S4.** Complete WD-XRF major- and minor-element compositions (wt.%) for all 21 samples, including elements not shown in main-text Table 2 ($P_2O_5$, Cl) and the unanalysed balance ("Other"). Values are semi-quantitative (no certified reference materials; no replicates).

| Sample | $SiO_2$ | $Al_2O_3$ | $Fe_2O_3$ | CaO | $K_2O$ | MgO | $Na_2O$ | $TiO_2$ | $P_2O_5$ | $SO_3$ | Cl | Other |
|---|---|---|---|---|---|---|---|---|---|---|---|---|
| GW-M1 | 60.28 | 14.54 | 5.00 | 10.26 | 2.44 | 4.12 | 2.29 | 0.62 | n.d. | n.d. | n.d. | 0.45 |
| GW-M2 | 61.34 | 15.08 | 5.05 | 8.33 | 2.36 | 3.54 | 2.16 | n.d. | 0.96 | n.d. | n.d. | 1.18 |

| | | | | | | | | | | | | |
|---|---|---|---|---|---|---|---|---|---|---|---|---|
| GW-M3 | 57.54 | 14.74 | 5.05 | 10.12 | 2.52 | 4.59 | 2.52 | n.d. | n.d. | 0.89 | n.d. | 2.03 |
| GW-M4 | 67.21 | 17.23 | 4.78 | 2.23 | 2.43 | 2.68 | 2.13 | 0.78 | n.d. | n.d. | n.d. | 0.53 |
| GW-Q1 | 66.47 | 17.86 | 5.27 | 1.99 | 2.77 | 2.63 | 1.89 | 0.79 | n.d. | n.d. | n.d. | 0.33 |
| GW-Q2 | 66.64 | 17.96 | 5.34 | 1.56 | 2.62 | 2.75 | 1.87 | 0.74 | n.d. | n.d. | n.d. | 0.52 |
| GW-Q3 | 69.27 | 16.45 | 5.06 | 1.00 | 2.48 | 2.25 | 2.21 | 0.81 | n.d. | n.d. | n.d. | 0.47 |
| GW-Q4 | 65.81 | 18.52 | 5.32 | 1.57 | 2.88 | 2.52 | 2.15 | 0.80 | n.d. | n.d. | n.d. | 0.43 |
| LY-T1 | 64.30 | 16.62 | 5.65 | 5.27 | 2.71 | 2.56 | 1.65 | 0.68 | n.d. | n.d. | n.d. | 0.56 |
| LY-T2 | 66.51 | 15.55 | 5.09 | 4.29 | 2.24 | 2.07 | 3.04 | 0.68 | n.d. | n.d. | n.d. | 0.53 |
| LY-TWD | 55.26 | 14.15 | 4.99 | 10.21 | 2.72 | 3.65 | 3.76 | n.d. | n.d. | 2.98 | n.d. | 2.28 |
| LY-DXB | 57.99 | 15.06 | 5.45 | 7.97 | 2.42 | 3.36 | 4.60 | n.d. | n.d. | n.d. | 1.67 | 1.48 |
| ZG-1 | 62.47 | 14.99 | 4.93 | 8.20 | 2.48 | 3.48 | 1.93 | 0.73 | n.d. | n.d. | n.d. | 0.79 |
| ZG-2 | 64.25 | 15.39 | 4.80 | 4.13 | 2.50 | 4.06 | 1.96 | 0.84 | n.d. | n.d. | n.d. | 2.07 |
| ZG-3 | 66.15 | 15.94 | 4.94 | 3.49 | 2.70 | 3.00 | 2.08 | 0.70 | n.d. | n.d. | n.d. | 1.00 |
| ZG-4 | 61.10 | 14.55 | 4.98 | 9.16 | 2.56 | 4.14 | 1.85 | 0.70 | n.d. | n.d. | n.d. | 0.96 |
| ZG-5 | 63.45 | 15.08 | 5.07 | 3.92 | 2.95 | 4.87 | 2.19 | n.d. | n.d. | 0.63 | n.d. | 1.84 |
| WP-SE2 | 53.60 | 14.17 | 5.47 | 7.04 | 2.36 | 6.19 | 2.25 | 0.82 | n.d. | 7.21 | 0.43 | 0.46 |
| WP-SE3 | 39.79 | 12.27 | 4.72 | 20.24 | 1.69 | 6.05 | 2.40 | 0.55 | n.d. | 9.82 | 2.10 | 0.37 |
| WP-SE4 | 63.66 | 15.22 | 4.67 | 2.79 | 2.68 | 6.16 | 2.11 | 0.74 | 0.37 | 1.21 | n.d. | 0.39 |
| WP-NE5 | 65.15 | 17.08 | 5.30 | 2.37 | 2.73 | 3.71 | 2.03 | 0.70 | 0.27 | 0.35 | n.d. | 0.31 |

**Note:** n.d. = not detected or below the quantification limit. "Other" is a residual balance calculated as 100% minus the listed oxides and minor elements (ranging from 0.31 to 2.28 wt.%) and may include volatile components ($H_2O$, $CO_2$, $SO_2$), unreported trace elements below quantification, and analytical uncertainty. Because loss on ignition (LOI) was not measured independently, this residual should not be interpreted quantitatively as LOI. Analysis: Panalytical Axios WD-XRF, Omnian semi-quantitative fundamental-parameter procedure.

**Table S5.** Principal component loadings (z-score-standardized PCA on six oxides; primary model in the main text) and variance explained.

| Oxide (clustering variable) | PC1 loading | PC2 loading |
|---|---|---|
| $SiO_2$ | +0.470 | +0.300 |
| $Al_2O_3$ | +0.472 | -0.035 |
| $Fe_2O_3$ | +0.222 | -0.919 |
| CaO | -0.469 | -0.213 |
| $K_2O$ | +0.382 | +0.066 |
| MgO | -0.376 | +0.121 |

**Note:** Variance explained: PC1 = 64.7%, PC2 = 15.3% (z-score model). PC1 separates silico-aluminous (high $SiO_2$, $Al_2O_3$, $K_2O$) from calcareous/ferromagnesian (high CaO, MgO) compositions; PC2 is dominated by $Fe_2O_3$. Under the CLR-plus-standardization sensitivity model, PC1 explains 81.6% of the variance (Table S2). K-means clustering used the z-scored six-oxide matrix (n_init = 50, random_state = 42).

**Table S6.** K-means cluster assignments per sample (k = 2 and k = 4) together with the XRD assemblage type, as summarized analytically in main-text Table 7.

| Sample | XRD type | k = 2 cluster | k = 4 type |
|---|---|---|---|
| GW-M1 | A | A (calcareous) | I |
| GW-M2 | A | A (calcareous) | I |
| GW-M3 | A | A (calcareous) | I |
| GW-M4 | A† | B (non-calcareous) | II |
| GW-Q1 | B | B (non-calcareous) | IV |
| GW-Q2 | B | B (non-calcareous) | IV |
| GW-Q3 | B | B (non-calcareous) | II |
| GW-Q4 | B | B (non-calcareous) | IV |
| LY-T1 | B | B (non-calcareous) | IV |
| LY-T2 | B | B (non-calcareous) | II |
| LY-TWD | B | A (calcareous) | I |
| LY-DXB | B | A (calcareous) | I |
| ZG-1 | B | A (calcareous) | I |
| ZG-2 | B | A (calcareous) | II |
| ZG-3 | B | B (non-calcareous) | II |
| ZG-4 | B | A (calcareous) | I |
| ZG-5 | B | A (calcareous) | I |
| WP-SE2 | B | A (calcareous) | I |
| WP-SE3 | B* | A (calcareous) | III (outlier) |
| WP-SE4 | B | A (calcareous) | I |
| WP-NE5 | B | B (non-calcareous) | IV |

**Note:** The k = 2 cluster is the two-group K-means partition on six z-scored oxides (not a CaO threshold). XRD type denotes the dominant feldspar species (A = anorthite-bearing; B = albite-bearing; B* = albite-bearing with secondary gypsum) and does not by itself indicate bulk CaO content. † GW-M4 retains the anorthite-bearing (Type A) assemblage assigned by the original XRD analysis but is a compositional (non-calcareous) outlier that groups with the non-calcareous cluster; this chemistry–mineralogy discrepancy is interpreted cautiously (see main text).
